# Supplementary figures and images for: Circulating Levels of Branched-Chain Amino Acids Are Associated with Diet: A Cross-Sectional Analysis
Source: Nutrients. 2025 Apr 27;17(9):1471. doi: 10.3390/nu17091471 (PMC12073366; doi:10.3390/nu17091471)

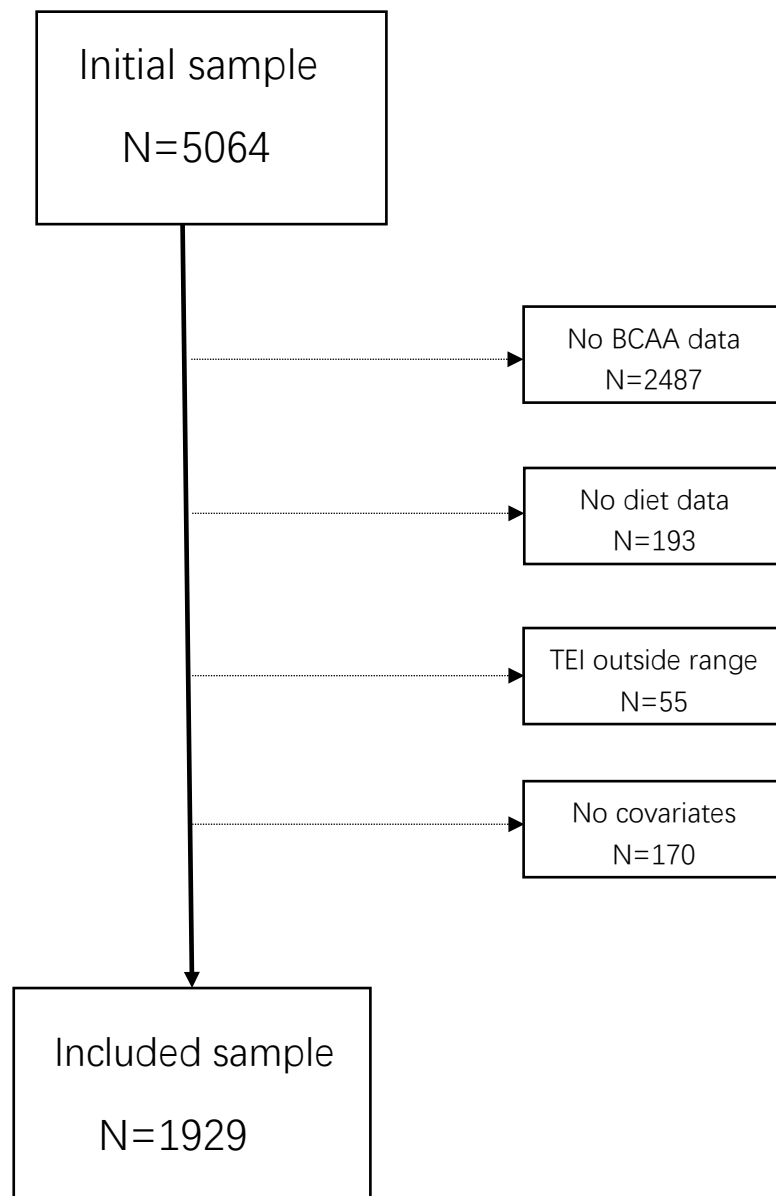

**Figure S1.** Selection procedure, CoLaus|PsyCoLaus study, Lausanne, Switzerland.

Supplement: Supplementary file 1 [file nutrients-17-01471-s001.zip › supplementary figure S1.pdf]
